# Correction Factors for Sclerometric Test Results in the Technical Assessment of Timber Structural Elements under Diverse Conditions

**DOI:** 10.3390/ma16247582

**Published:** 2023-12-10

**Authors:** Justyna Jaskowska-Lemańska, Daniel Wałach, Monika Górka-Stańczyk

**Affiliations:** 1 Faculty of Civil Engineering and Resource Management, AGH University of Krakow, Adama Mickiewicza Ave. 30, 30-059 Krakow, Poland; 2 Faculty of Civil Engineering, Cracow University of Technology, Warszawska 24, 31-155 Krakow, Poland

**Keywords:** timber structures, semi-destructive tests, sclerometric tests, pin penetration test, technical condition assessment, relation to annual growth rings, temperature

## Abstract

Research on existing wooden structures relies on non-destructive and semi-destructive techniques. One of the methods enabling the estimation of the physico-mechanical characteristics of wood in building structures based on established correlational relationships is the sclerometric method. The challenge in utilizing these known correlational relationships is the lack of data regarding the impact of frequently occurring factors in objects on sclerometric test results. This paper presents the influence of selected factors on the results of sclerometric tests, such as temperature, the direction of testing in relation to annual growth rings, and the physical orientation of the measuring device. The research was conducted on pine, spruce, and fir elements, each subjected exclusively to the influence of one of these factors. The study indicates that these factors should not be overlooked in assessing technical conditions using sclerometric testing methods. The impact of temperature on sclerometric test results is relatively small; a change in temperature of 10 °C results in an average test outcome change of approximately 3%. Conversely, changing the orientation of the measuring device from horizontal to vertical can alter the test result by up to 10%. The direction of testing relative to the annual increments of wood also has a significant impact on the test results, but incorporating this factor into practice seems to be quite difficult, and in the case of elements with substantial cross-sections, it is also not required. The obtained results enable the application of established correlational relationships in the structural analysis of wooden elements for which access is challenging, especially under temperature conditions different from the reference, 20 °C.

## 1. Introduction

Non-destructive and semi-destructive testing of wooden structures has become increasingly prevalent in recent years in the fields of civil engineering and historic preservation [1,2,3,4]. The increasing significance of these examinations stems from technological advancements, ensuring the continuous evolution and widespread availability of employed devices, and a growing environmental awareness. These methods are perceived as sustainable and environmentally friendly as they can minimize waste generation and material consumption [5,6]. In the field of historic preservation, these methods also represent an indispensable tool that allows for the assessment of the technical condition of construction elements without the need for their disassembly or destruction, especially where preserving the original structure is a priority. Furthermore, the standards established in 2019 [7,8] acknowledge non-destructive (NDT) and semi-destructive (SDT) methods as significant tools for assessing the technical conditions of historic structures. However, these standards primarily offer a framework of general guidelines for testing, proposing potential methods while delegating the specifics of their application to the judgment of experts. In contrast to the comprehensive methodologies typically prescribed for testing materials such as concrete, these guidelines do not provide detailed procedures for assessing historic wooden structures.

One of the semi-destructive methods, which is relatively simple and economical to use and whose results can form the basis for determining the physico-mechanical properties, is sclerometric testing [9]. These tests are well-known and extensively documented in the literature in the context of assessing concrete elements [10]. However, when it comes to sclerometric testing for wooden elements, there is still a lack of sufficient research and analysis to enable their broader application. Primarily known are the numerical influences for other non-destructive and semi-destructive wood tests, especially acoustic testing [11,12], while in the case of sclerometric testing, they are most often limited to describing the impact of moisture on their results [13,14,15].

It is important to note that the investigation of existing wooden structures is associated with various challenges arising from factors such as access to the element, its current load level, moisture content [16], age [17], degradation, and the condition of the surface [4,18]. In light of the utilization of various characteristics of wood as a material in construction, we will discuss elements subjected to bending, compression, or tension. These elements, depending on their location within a structure, can be examined on all surfaces or only in selected areas and specific directions. The direction of the examination can be considered in two variants: the physical direction of the test (vertically upwards or downwards, horizontally, or diagonally) and the direction of the test relative to the annual growth rings of the wood.

Wood displays orthotropic characteristics, meaning its physical and mechanical properties vary along three orthogonal axes: longitudinal (along the grain), radial (across the grain, from the center outwards), and tangential (perpendicular to the radial direction, along the growth rings). This orthotropy arises from the wood’s natural growth structure, where the alignment of cellulose fibers and the arrangement of vascular bundles create distinct mechanical behaviors in each direction. Incorporating this orthotropic model into testing methods is crucial for enhancing the accuracy and reliability of assessments in wood materials, ensuring a more comprehensive understanding of their condition and structural integrity. This aspect is extensively investigated in ultrasonic testing, where results can vary significantly in different directions [19]. Similarly, in semi-destructive testing, such as drilling resistance tests, changing the test direction from radial to tangential noticeably influences outcomes [20]. Yet, there is a notable gap in research quantifying how test direction affects sclerometric test results in various wood species with differing densities.

In structural engineering, the feasibility of non-destructive or semi-destructive testing on wooden structural elements like rafters, floor beams, and columns is largely dictated by their placement within a construction. The orientation of these elements primarily determines the possible testing directions, influenced by their integration into the structure and often restricting access to certain faces. This directional preference is a direct consequence of the way these elements are embedded in the structure, often limiting the accessibility to certain faces or sides. Additionally, the dimensions of the wooden element significantly influence the choice of testing direction. For instance, in larger beams, the tangential direction might be more accessible, whereas in slender columns, the radial direction could be more feasible for testing. This interplay between the structural role, orientation, and dimensions of wooden elements is critical in planning and executing effective non-destructive or semi-destructive testing strategies, ensuring an accurate assessment of the wood’s integrity and performance within the structure.

Moreover, these examinations will not always be conducted on elements with the same temperature or moisture content [21,22,23]. Until now, correlational relationships have been presented as those determined for reference conditions—most commonly at 20 °C and 12–15% moisture content. These studies present correlational curves for such wood species as pine, spruce, fir [24,25,26,27] and chestnut [28,29] originating from various habitats across Europe. Both in the literature and in materials provided by manufacturers, there is largely a lack of a uniform research procedure that would indicate how to take into account selected factors in the results of sclerometric tests.

This paper presents the results of research on the influence of selected factors in the context of sclerometric testing performed with the WoodTester device for pine, spruce, and fir wood, in the form of correction indices that can be applied to account for conditions other than comparative testing conditions. The study analyzed the impact of test multiplicity, the direction of testing relative to annual growth rings, and the influence of temperature.

## 2. Materials and Methods

For the research, a diverse range of pine (*Pinus sylvestris*), spruce (*Picea abies*), and fir (*Abies alba*) wood from the Central European region was collected. The size of the samples was adjusted depending on the factor being analyzed. All tests were conducted using the WoodTester Novatest device (Ancona, Italy). The test involves driving a steel needle into the wood with a constant impact energy of 2.4 J, positioning the device perpendicular to the material’s surface. The tests were performed with steel needles dedicated to the device, 50 mm in length with a conical tip at a 35° angle and a Rockwell hardness of 60. In the conducted studies, the manufacturer’s instructions were adhered to, ensuring that the steel needles were spaced at least 25 mm apart from each other and from the edge of the element. Readings of the needle’s penetration depth were taken using a dial gauge sensor with an accuracy of 0.01 mm. The measured value represented the unpenetrated portion of the needle. The measurement result was calculated as the difference between the length of the needle and the measured size. The set used in the studies is shown in Figure 1.

To illustrate the nature of the damage caused by the test on selected samples, additional tomographic imaging was conducted using the GE Phoenix v-tome-x m device (Figure 2a). The study used samples approximately 15 mm × 15 mm × 40 mm in size, containing holes resulting from sclerometric tests (Figure 2b). X-ray images resulting from tomographic examination at the test sites (holes after needle penetration) are presented in the following sections: transverse within earlywood *R1*, visible tangential surface of the wood; transverse within latewood *R2*, visible tangential surface of the wood; longitudinal *T*, visible radial surface of the wood; longitudinal *L*, visible end surface of the wood.

### 2.1. Impact of the Testing Direction Relative to the Annual Growth Rings

To quantitatively determine the impact of the testing direction in sclerometric tests relative to the annual growth rings, a series of tests were conducted on 8 samples of each wood species (pine, spruce, and fir). The test elements, measuring 50 mm × 50 mm × 1000 mm, each had six research fields (Figure 3a) designated to eliminate the influence of potential wood defects (three fields on edges perpendicular to each other). The tests were conducted in four directions: perpendicular, parallel, and oblique (~30° and 60°), relative to the annual growth rings (Figure 3b,c). For each wood species, the samples were selected so that 4 of them allowed for perpendicular and parallel testing to the fibers and 4 at oblique angles of approximately 30° and 60°. In each field, at least 6 points were designated, spaced at least 25 mm apart from each other and from the edge of the element. Consideration was given to the needle penetration depth after both single and double hammer impacts.

For each species, high-quality samples (with a minimum number of natural wood defects) were chosen with closely matched density and similar widths of annual growth rings; although, for fir wood samples, the average ring width was greater than in other species. The moisture content of the samples at the time of the sclerometric testing was 12 ± 1%.

### 2.2. Impact of the Orientation of the Measuring Device and the Multiplicity of Impacts

The impact of the orientation of the measuring device on test results was determined using six fir wood elements with varied bulk densities: two elements, each at densities of approximately 380, 480, and 580 kg/m^3^. The test elements measured 60 mm × 300 mm × 550 mm. The tests were conducted perpendicular to the annual growth rings. Measurement fields for tests in each of the considered directions were designated on each sample, maintaining a distance of at least 25 mm between measurements and from the edge of the element. In each direction (horizontal testing and vertical testing ‘downwards’ and ‘upwards’), 18 measurements of single and double penetration were performed (Figure 4). The moisture content of the samples at the time of the sclerometric testing was 12 ± 1%.

On the same sample, to determine the impact of the number of impacts on the results obtained, additional tests were conducted. For the test with the measuring device in a horizontal position, successive impacts were performed for each point, obtaining results from single to quintuple impacts for the three analyzed material densities.

### 2.3. Impact of Temperature on Test Results

The assessment of the impact of the temperature of wooden elements on sclerometric test results was conducted within the range of possible conditions of structural use: −20 °C, 0 °C, + 20 °C, and + 50 °C. The prepared research program reflects the following scenarios: an examination of unheated buildings (e.g., roof trusses) during exceptional frosts, a standard winter, normal conditions, and a hot summer.

The tests were carried out on 8 samples of each wood species (pine, spruce, and fir). The test elements measured 175 mm × 175 mm × 300 mm. Two fields were designated on each sample, and in each field, at least 18 points were set apart from each other and from the edge of the element by at least 25 mm (a minimum of 6 points for each temperature level). Consideration was given to needle penetration after a single and double hammer impact. The layout of the tested sample is shown in Figure 5.

The samples were divided into two groups: a temperature-lowering group from +20 °C to −20 °C (four samples of each species) and a temperature-raising group from +20 °C to +50 °C (four samples of each species). All temperature changes were made while maintaining a moisture content of 12 ± 1% (this was the air-dry state). After the reference test in the air-dry state at approximately +20 °C, the first group was tightly sealed with PE film and placed in a freezing chamber for 72 h at 0 °C. Following this, the film was removed, and a series of tests were performed while maintaining the temperature. The procedure was then repeated at −20 °C. The second group of samples, after the reference test in the air-dry state at +20 °C, was tightly sealed with PE film and subjected to heating processes at +50 °C in laboratory dryers without air circulation. The heating process lasted 8 h, with the first three hours reaching the target temperature. The samples were tested immediately after removal from the dryer and the film.

## 3. Results

### 3.1. Research on the Direction Relative to Annual Growth Rings

A comparison of the sclerometer needle penetration depth for samples tested in perpendicular, parallel, and oblique directions relative to the annual growth rings is shown in Figure 6 (for a single impact) and Figure 7 (for a double impact). The box plot represents 25 ÷ 75%, with the median line indicating the average value and the whiskers representing the minimum and maximum values. Detailed data are presented in Appendix A, Table A1.

The depth of penetration of the sclerometer needle is dependent on the direction of testing relative to the annual growth rings. Generally, greater average penetration is observed for smaller angles of testing relative to the growth rings (0° and 30°) and lower values for larger angles (60° and 90°), both for single and double impacts. In comparison with testing at a right angle to the growth rings, the largest decrease in needle penetration depth was observed for spruce wood with a double impact (3.3%), while the greatest increase in depth was recorded for pine and fir wood with a single impact (~14%). The most uniform results were obtained for tests perpendicular to the growth rings, whereas the greatest variation in test results was observed in the direction parallel to the growth rings, particularly for pine and spruce wood and at 30° for fir wood. Table 1 presents the percentage change in the average value result depending on the wood species, assuming that the reference value is the test at a right angle to the fibers.

Figure 8, Figure 9 and Figure 10 present X-ray images of the wood destruction structure that occurred during sclerometric testing in perpendicular, parallel, and oblique directions to the annual growth rings. It is evident that the character of microstructure destruction varies both within the early and late wood of the same sample and across different testing directions relative to the annual growth rings.

The passage of the sclerometer needle for tests perpendicular to the growth rings causes both elastic deformation and permanent deformation of individual wood fibers. This destruction is accompanied by the breaking of the structure and brittle fractures of the fibers, especially in the late wood, which manifests as an increase in the destruction zone in the longitudinal direction. These cracks are significantly larger than the diameter of the sclerometer needle, averaging up to 2.8 mm from the hole’s axis. In Figure 8a,b, the damaged fibers are marked with a black dashed line. A significant expansion of this area within the late wood is visible. The cross-section of the sclerometer needle is shown in orange in Figure 8a,b. On the end and radial surfaces of the wood (Figure 8c,d), along the axis of the hole, we observe the extent of the destruction of the wood fibers and the accumulation of chips in the lower part of the hole.

In the analyzed scan (Figure 9), the sclerometer needle’s passage in the test parallel to the annual growth rings predominantly involves penetration within the summer growth (early wood). The destruction is accompanied by a definitive breaking of the structure within the early wood and partially elastic behavior in the late wood (the cross-sectional diameter of the hole is smaller than the diameter of the sclerometer needle). In Figure 9a,b, the damaged fibers are indicated with black dashed lines. In this case, an expansion of this area in the longitudinal direction is visible. On the end surface, we can observe the destruction of the structure within the adjacent summer growths (a step towards the axis of the hole). On the end and tangential surfaces of the wood along the axis of the hole, we observe the extent of the destruction of the wood fibers and the accumulation of chips in the lower part of the hole. The extent of destruction, as depicted, will vary depending on the width of the annual growth rings and the position of the hole’s axis (whether in summer or winter growth).

Figure 10 displays tomographic images from sclerometric tests conducted at an angle of approximately 60° to the annual growth rings. The passage of the sclerometer needle in tests at this angle demonstrates characteristics akin to those in right-angle tests but involves a significantly greater extent of brittle destruction along the fibers (averaging 3.6 mm from the hole’s axis in the late wood zone). A slight elastic deformation is observable in the transverse direction, with the remaining hole after testing in this direction being about 0.2 mm smaller than the diameter of the sclerometer needle. As with Figure 8 and Figure 9, we observe an accumulation of wood chips in the lower part of the hole and the ‘pulling’ of wood fibers consistent with the direction of needle penetration. Testing at an angle to the growth rings demonstrates the greatest destruction within the wood’s microstructures. The image of such destruction, like the impact of direction on test results, will undoubtedly depend on the angle of inclination to the growth rings (closer to perpendicular or parallel).

### 3.2. Research on the Physical Direction of Multiple Impact Testing

The results vary for different orientations of the device relative to the tested element, attributable to the nature of the measurement, in which the weight allowing for the generation of the appropriate energy mounted in the device plays a significant role, similar to sclerometric testing for concrete. Thus, when the device is oriented horizontally, the force of gravity acts perpendicularly to the direction of the force driving the needle. In contrast, for vertical needle penetration upwards or downwards, the direction of gravity’s effect on the weight aligns with the needle-driving force, and additionally, their directions differ for downward penetration. The study results are presented in Figure 11. The box is represented as 25 ÷ 75%, with the median line indicating the average value and the whiskers representing the minimum and maximum values. Detailed data are presented in Appendix A, Table A2.

The general trend, irrespective of material density and number of impacts, shows increased needle penetration depth in the ‘vertically downward’ orientation and decreased depth in the ‘vertically upward’ orientation, compared to horizontal positioning.

The extent of this impact is slightly dependent on material density (within the tested range). For a single impact, the differences between horizontal testing and vertical down testing were similar, at approximately 7%. In contrast, the differences for vertical up testing relative to horizontal positioning were approximately 6%. For a double impact, when comparing horizontal orientation with vertical down orientation, the differences increased to around 10%, while with the vertical up orientation, the differences generally decreased. Table 2 compiles the percentage change in the test result performed with different device orientations for elements of varying densities. The baseline device position was taken as horizontal and perpendicular to the surface of the element, which is typically the orientation for which correlational relationships are presented.

For the same sample, the impact of the number of impacts on the uniformity of results was determined. The results, regardless of the number of impacts, confirm the generally known relationship: the greater the density of the material, the lesser the penetration depth. However, they do not support the hypothesis that analyzing a larger number of annual growth rings caused by deeper needle penetration with multiple impacts leads to greater statistical uniformity. Figure 12 presents the results of these studies, using a box to represent the 25 ÷ 75% with the median line indicating the average value and whiskers representing the minimum and maximum values. Detailed data are provided in Appendix A, Table A3.

With an increasing number of indentations, a greater dispersion of results is observed, along with an overlap of result intervals (represented by the whiskers) for higher numbers of impacts. Within the results for single and double impacts, there is no overlap of maximum results with the minimum of the subsequent impact. In the case of three, four, and five impacts, the maximum results reached the interval values of subsequent impacts, particularly in higher densities (480 ÷ 580 kg/m^3^). Comparing single and double impacts, greater differences were seen between the maximum and minimum values obtained among the different densities for the double impact relative to the single impact. Based on these results, it can be concluded that a greater number of impacts does not lead to increased measurement accuracy. Table 3 compiles the percentage changes in results for subsequent needle penetration impacts of the sclerometer.

### 3.3. Impact of Temperature on Test Results

A comparison of average needle penetration depths of the sclerometer for samples tested at temperatures ranging from −20 °C to + 50 °C, along with the designated trend line and corresponding Pearson correlation coefficients r, is presented in Figure 13. Detailed data are provided in Appendix A, Table A4.

For all wood species studied, the depth of penetration of the sclerometer needle depends on the temperature of the tested sample. Generally, a greater average needle penetration is observed at higher temperatures and less penetration at lower temperatures; these changes within this temperature range can be described as linear. The most significant differences were observed in pine samples, with an average decrease in penetration of 11.5% at −20 °C and an average increase of 7.5% at +50 °C. In the case of fir wood samples, the impact of temperature on test results was lower. The decrease in penetration depth at −20 °C averaged 3.7% relative to the reference test, while the increase in penetration depth with a temperature rise to +50 °C averaged 5.1%. Table 4 compiles the percentage change in penetration depth, taking +20 °C as the reference level, and the average percentage change per 10 °C temperature change is calculated based on the obtained linear correlations.

## 4. Discussion

### 4.1. Impact of Testing Direction Relative to Annual Growth Rings on Obtained Results

Most commonly, in assessing the technical condition of large-dimension wooden structural elements (such as floor beams), we will encounter tests conducted perpendicular to the annual growth rings. However, in the case of slender elements (such as modern rafters or beams of frame structures), the direction of testing relative to the growth rings can also be oblique or close to parallel to the annual growth rings [30].

The analysis indicates that the testing direction relative to the annual growth rings significantly impacts sclerometric test results in wooden structures and should not be overlooked. Discrepancies in the results of tests conducted in various directions relative to the growth rings reach approximately 14%. In relation to other NDT and SDT studies, the existing literature corroborates the importance of this factor [20,28,31]. Sclerometric test results not only depend on the direction of testing relative to the growth rings but also on their width. Consequently, these tests exhibited relatively large variances in outcomes. The largest decrease in penetration depth, regardless of the wood species, was obtained for tests conducted parallel to the growth rings. This is directly related to the microscopic structure of wood. Testing parallel to the growth rings allows for the destruction of the weaker early wood structure both in the tested zone and in the adjacent growths without significantly disrupting the structure of the late wood beyond the immediate penetration zone of the sclerometer needle. This pattern of destruction means that with the same impact energy, more early wood structures are destroyed, allowing the sclerometer needle to penetrate much deeper. Ideally, one would expect that for tests at angles of 30° and 60°, there would be a reduction in needle penetration due to its longer path within the denser late wood than for tests at a right angle. This result was obtained only for the 60° angle test, although this reduction is not particularly large, and for fir wood, which had larger ring patterns, the change is practically negligible. In contrast, for the 30° angle tests, an increase in penetration was observed, which may result, similar to tests parallel to the growth rings, from the impact of the sclerometer’s impact force on the early wood. It is worth noting that the impact of the sclerometric testing direction relative to the annual growth rings is generally less for double-impact testing than for single-impact testing. The observed variations in sclerometric test results, as influenced by the testing direction relative to the annual growth rings in wooden structures, can be largely attributed to the inherent physical properties of wood. Wood, being an anisotropic material, exhibits varying properties depending on the direction relative to its grain. In sclerometric testing conducted perpendicular to the growth rings, the needle encounters a more homogenous structure, resulting in more consistent penetration depths. However, testing parallel to the growth rings predominantly involves the early wood, which is typically softer and less dense than the late wood. This interaction results in deeper penetration due to the destruction of the weaker early wood structure without significantly affecting the denser late wood. At oblique angles, such as 30° and 60°, the needle traverses both early and late wood, yielding variable results due to differing densities. These findings highlight the complex interaction between the sclerometer needle and the wood’s microstructure, which varies with the angle of impact. The discrepancy between single and double-impact testing outcomes underscores the sclerometric method’s sensitivity to specific testing conditions.

The conducted studies were carried out on specially selected samples allowing measurement in one particular direction relative to the growth rings. In practice, such a situation will occur relatively rarely and will mainly concern elements with small cross-sectional dimensions.

Practically, for elements with a longer history of use, where the wood grain pattern (which would at least reveal the location of the tangential plane) is not visible, it seems challenging to unequivocally determine the orientation of the device relative to the annual growth rings for testing. In such structures, it is unlikely that we will only encounter testing exclusively in perpendicular or parallel directions to the growth rings. More often, the angles will be intermediate. In such cases, using correction factors for this specific factor seems unjustified, especially in sclerometric tests involving double impacts.

In assessing contemporary structures using sclerometric methods, the wood grain pattern should typically allow for positioning the device in relation to the annual growth rings and determining their approximate width. In such applications, the use of Formula (1) along with coefficients compiled in Table 5 is proposed. It is assumed that PDx_90_ is the result of a single or double sclerometric test for perpendicular orientation to the growth rings, presented in millimeters, PD_r_ is the result of a given sclerometric test at a given angle relative to the growth rings, and r is the coefficient relating to the percentage change in the result. For wide annual growth rings, the selection of maximum values is proposed, and for very narrow rings, the minimum values. In other cases, the use of the average value is recommended.

PD_x90_ = PD_r_ [1 + r]. (1)

### 4.2. Research on the Impact of Physical Testing Direction and Multiplicity

Regarding the physical direction of wood testing in the case of sclerometric tests, there is a justified concern that it will have a significant impact on the results. This is related to the construction and operating principle of the sclerometer, similar to what is observed in the sclerometric testing of concrete [10]. By default, these tests are performed in a horizontal orientation, yet this positioning may not always be feasible due to the specific nature of the structure. Analysis reveals that the physical orientation of the device relative to the tested element significantly influences the testing outcome. Given the minimal variation in impact across different material densities, it is suggested for engineering purposes to utilize a correction coefficient as outlined in Formula (2). In this context, PD_x_ refers to the outcome of either a single (PD_1_) or double (PD_2_) sclerometric test with the device in a horizontal position, measured in millimeters. PD_p_ denotes the result of the test in a vertical orientation, either upwards or downwards, and p represents the coefficient indicating the percentage change in results, as specified in Table 6.

PD_x_ = PD_p_ [1 + p]. (2)

The manufacturer’s instructions for the Woodtester Novatest device suggest 5-fold needle penetration before making a measurement. However, in the case of testing softwood, such as coniferous wood, this recommendation may not always be feasible (at lower densities, full possible penetration can be achieved by around four impacts). It should be noted that analyzing a larger number of annual growth rings caused by deeper needle penetration with multiple impacts does not lead to greater statistical uniformity.

The outcome of the sclerometric test varies almost proportionately to the number of penetrations, exhibiting a marginally smaller percentage difference at higher densities. This is directly related to the nature of the measurement, which is strongly dependent on microscopic structure and, thus, density. The increase in penetration depth tends to diminish with the number of impacts, likely attributable to the increasing friction on the side of the sclerometer needle. Formula (3) facilitates the approximate conversion of the *PD*_1_ value, expressed in millimeters, to the PD_x_ value obtained from two (PD_2_) to five (PD_5_) impacts, applicable for material densities ranging from 380 ÷ 580 kg/m^3^. The formula requires the use of a correction coefficient m presented in Table 7. Similar calculations, taking into account the percentage change in penetration depth with varying numbers of impacts, can be performed as outlined in Table 3.

PD_x_ = PD_1_ [1 + m]. (3)

### 4.3. Research on the Impact of Temperature

The results obtained indicate a relationship between temperature and the outcomes of sclerometric tests. Generally, the mechanical properties of wood decrease when it is heated and increase upon cooling [32,33]. Similarly, in other non-destructive and semi-destructive testing methods for wood, the impact of temperature on the results has been observed [34,35]. This tendency is also evident in sclerometric testing. Practically, the impact of temperature on wood’s mechanical properties is relatively minor. However, when assessing the technical condition under extreme conditions, it is important to account for the variations in measured quantities due to temperature changes, aligning the results with a reference temperature.

The most significant changes were noted in pine wood, whereas fir wood exhibited the least variation. In each case, however, a linear increase in the penetration depth of the sclerometer needle with increasing temperature was observed. The increase in the sclerometer needle’s penetration depth can be attributed to the rising temperature, causing wood fibers (cellulose) to move apart, reducing cohesion forces and, thereby, the mechanical strength of the wood. This effect is likely to be greater with higher wood density.

Due to the impact of temperature on sclerometric test results, it is recommended to perform these tests at a temperature close to the reference temperature (20 °C). In engineering applications where structures must be assessed using sclerometric methods at temperatures significantly deviating from the reference temperature (by more than 10 °C), the use of a corrective Formula 4 is proposed, along with coefficients compiled in Table 8. These allow for the conversion of the result of a single or double sclerometric test at temperatures ranging from −20 °C to + 50 °C (*PD_t_*) to a result defined at the reference temperature (*PD_t_*_=20_), assuming that the temperature difference Δt between the test and reference temperatures is expressed in Celsius degrees. Table 8 lists the minimum, maximum, and average values of the proposed correction coefficient u, corresponding to the percentage change in results. For wood with potentially high resin content, it is suggested to choose maximum values, and for completely resin-free wood, minimum values. In other cases, the use of the average value is recommended.

PD_t=20_ = PD_t_ [1 + u|Δt|]. (4)

## 5. Conclusions

The use of sclerometric methods in existing wooden structures focuses on the in situ estimation of the mechanical properties of structural dimension elements. Although there are known literature results for these tests applied to various wood species under laboratory conditions, it is difficult to extrapolate these results and conclusions to in situ measurements and assessments of existing structures. This challenge arises as these tests are typically conducted on small, defect-free samples or fresh structural wood, unaffected by various degrading factors (such as moisture or temperature) and where access to these elements was not additionally hindered (for example, by other structural or finishing elements).

This paper introduces a series of corrective coefficients, enabling the application of laboratory-established correlational relationships to in situ assessments of the technical condition of elements in different thermal-humidity and locational–geometrical conditions than the reference conditions. Certainly, conducting tests under conditions not requiring these coefficients is preferable, yet experience indicates that this is not always feasible. It is important to remember that these coefficients should be tailored to the specifics of the wood being tested, including its species, density, or resin content. Additionally, the presented coefficients were derived for selected factors; it is not determined whether the simultaneous influence of various factors will yield an effect corresponding to the cumulative consideration of the corrective coefficients.

Considering the guidelines set by the standard [7] for assessing the technical condition of wooden structures, researching the effective use of semi-destructive methods becomes imperative. In the case of sclerometric testing, this work appears to have addressed significant factors, yet further research should not be neglected. It should be noted that the microscopic structure of wood varies depending on its species and the region of harvest, leading to varying influences from individual factors. This variability may limit the universal applicability of the correction coefficients presented, akin to the correlation relationships documented in the literature. Beyond extending results to additional wood species from various regions, it will be crucial to determine a corrective coefficient for simultaneously changing moisture and temperature conditions. In analyzing various building structures, it is important to note that their availability for study is often significantly limited, and their load conditions can crucially influence the outcomes of sclerometric studies; thus, further research exploring the influence of stress states on these study results appears promising. In the case of historic structures, one can often encounter additional limitations, such as the presence of polychrome, which, on the one hand, can affect the results of the study and, on the other hand, significantly hinder the implementation of research work. One potential solution is the application of the active thermal imaging method [36]. In connection with this, as a further direction of research, we can indicate the analysis of the influence of finishing the element with paint layers as well as various types of surface treatment (e.g., drying and impregnation) on the research results. Future steps might interestingly involve research on the impact of stress conditions on these test results, as well as different types of surface treatments (e.g., drying and impregnation). Finally, an extremely interesting but also a challenging aspect to quantify is the impact of wood aging and degradation on the results of sclerometric testing.

One of the key limitations of sclerometric methods is their local nature, focusing on the surface structure of wood. It is crucial to acknowledge that wood, as a natural material, is susceptible to degradation processes which may not be immediately evident on the surface. For instance, internal degradation of beams embedded in walls can develop from the end grain side, remaining undetected during surface sclerometric examinations. This characteristic underscores the need for comprehensive assessment strategies when evaluating the condition of wooden structures involving a combination of diverse methods and techniques to mitigate the risk of inaccurate interpretations regarding the structure’s condition.

## Figures and Tables

**Figure 1:**
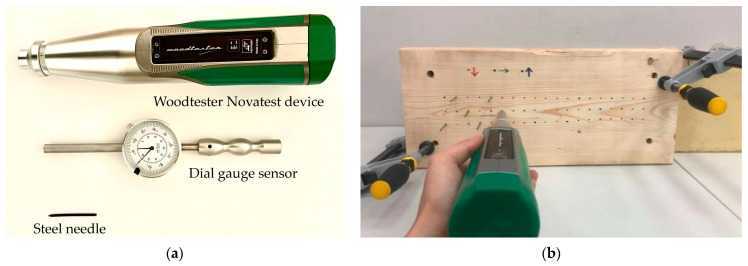
Woodtester Novatest (**a**) device set; (**b**) an example sample during the testing process.

**Figure 2:**
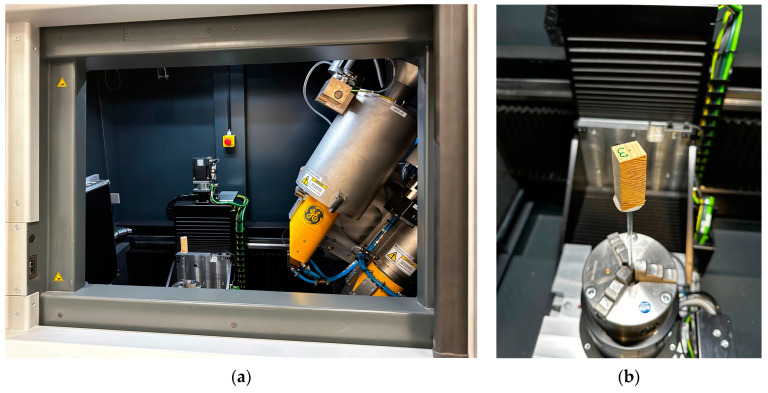
X-ray computed tomography studies: (**a**) GE Phoenix v-tome-x m device during the examination; (**b**) a sample prepared for tomographic studies mounted inside the device.

**Figure 3:**
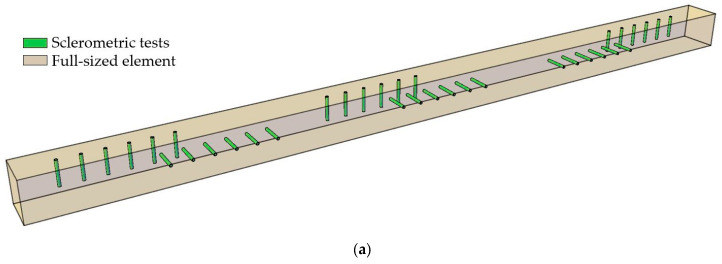

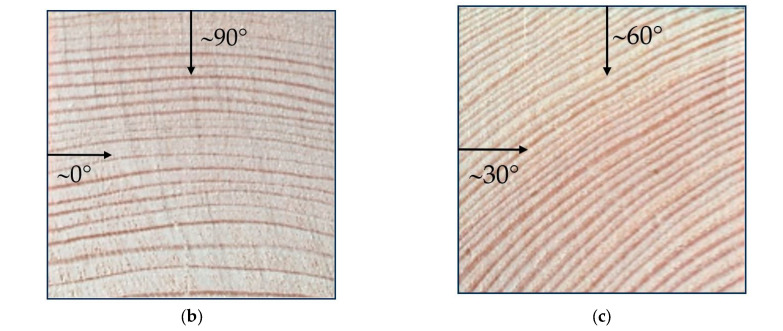
Sample used for studying the impact of the direction of annual growth rings on sclerometric test results: (**a**) scheme of the sample, (**b**) sample layout perpendicular and parallel, and (**c**) sample layout oblique (~30° and 60°).

**Figure 4:**
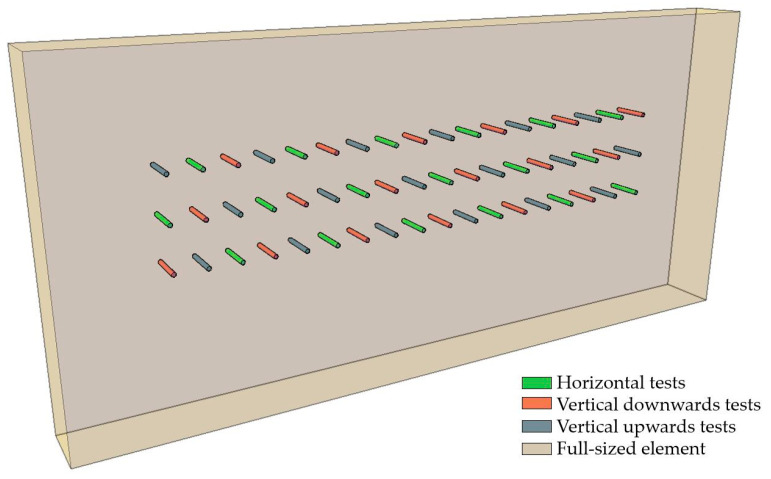
Scheme of sample used for studying the impact of device orientation.

**Figure 5:**
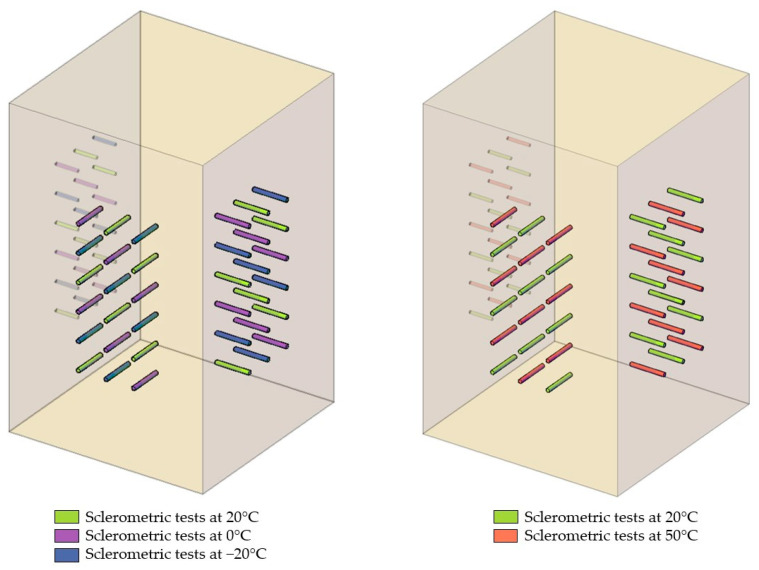
Scheme of sample used for studying the impact of temperature.

**Figure 6:**
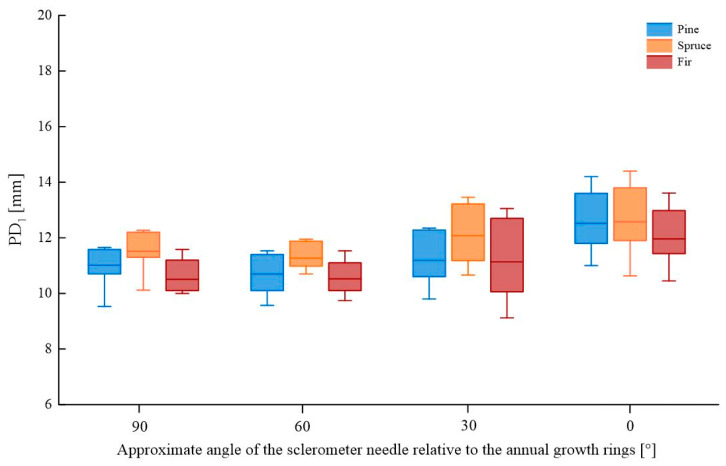
Comparison of sclerometric test results (single impact) for samples tested in various directions relative to annual growth rings.

**Figure 7:**
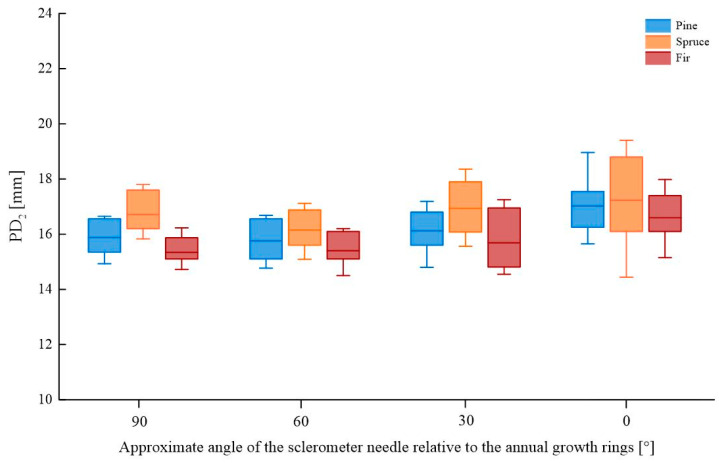
Comparison of sclerometric test results (double impact) for samples tested in various directions relative to annual growth rings.

**Figure 8:**
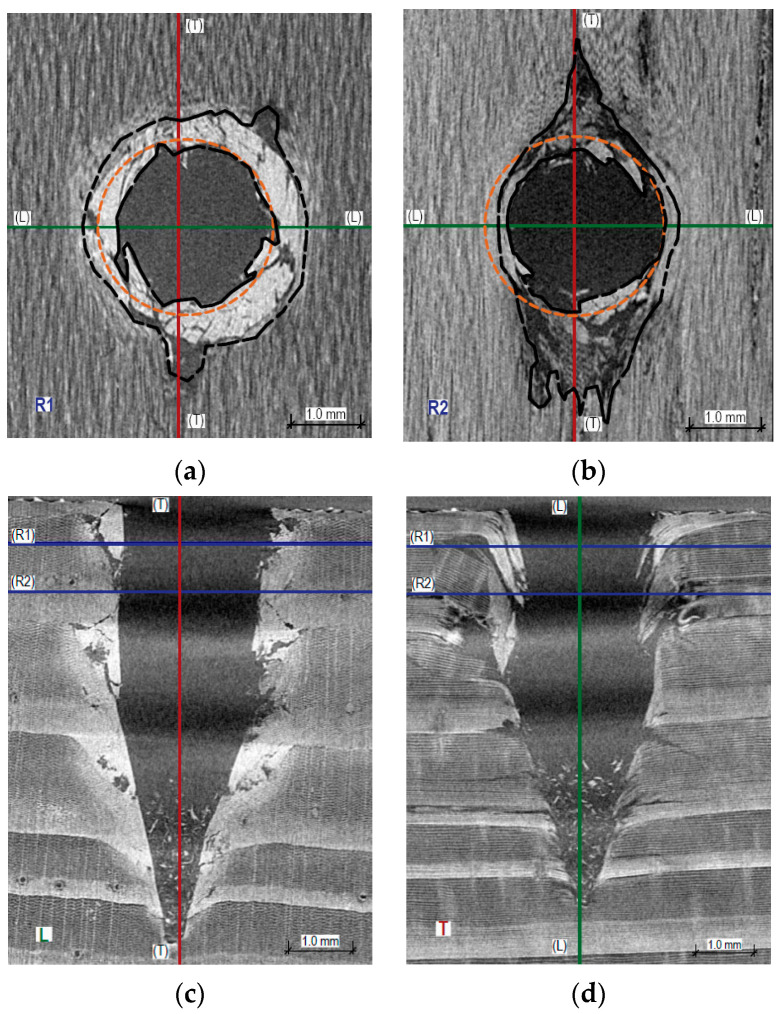
Cross-sections through the hole created by sclerometric testing (PD_1_) perpendicular to the annual growth rings: (**a**) cross-section within the earlywood R1, visible tangential surface of the wood; (**b**) cross-section within the latewood R2, visible tangential surface of the wood; (**c**) longitudinal section L, visible end surface of the wood; (**d**) longitudinal section T, visible radial surface of the wood.

**Figure 9:**
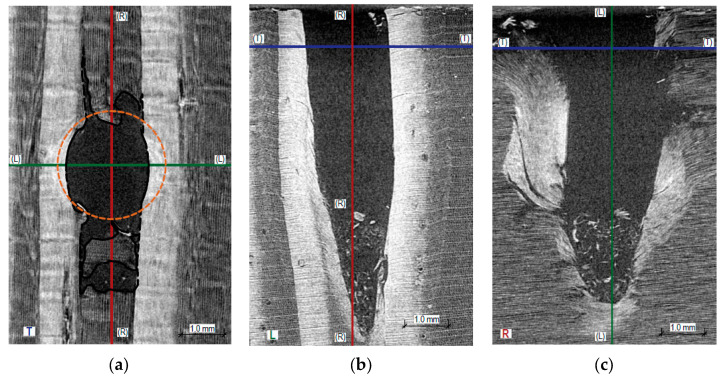
Cross-sections through the hole created by sclerometric testing (PD_1_) parallel to the annual growth rings: (**a**) transverse section T, visible radial surface of the wood; (**b**) longitudinal section L, visible end surface of the wood; (**c**) longitudinal section R, visible tangential surface of the wood.

**Figure 10:**
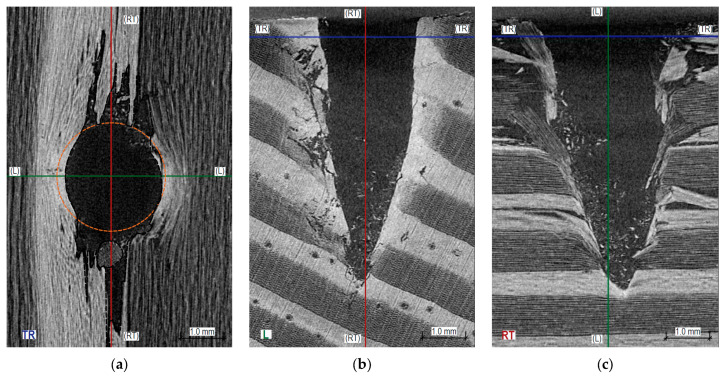
Cross-sections through the hole created by sclerometric testing (PD_1_) obliquely to the annual growth rings: (**a**) transverse–radial section TR, visible radial–tangential surface of the wood; (**b**) longitudinal section L, visible end surface of the wood; (**c**) radial–tangential section RT, visible radial–tangential surface of the wood.

**Figure 11:**
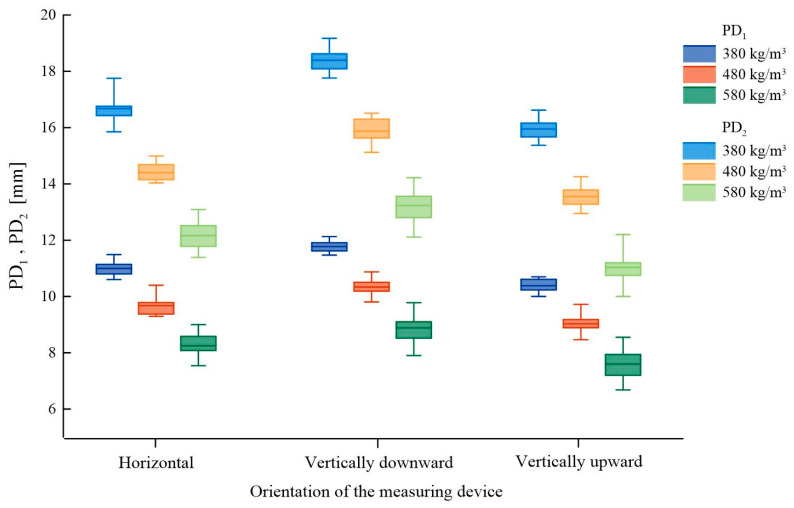
Impact of the orientation of the measuring device on sclerometric test results for samples with varied bulk density.

**Figure 12:**
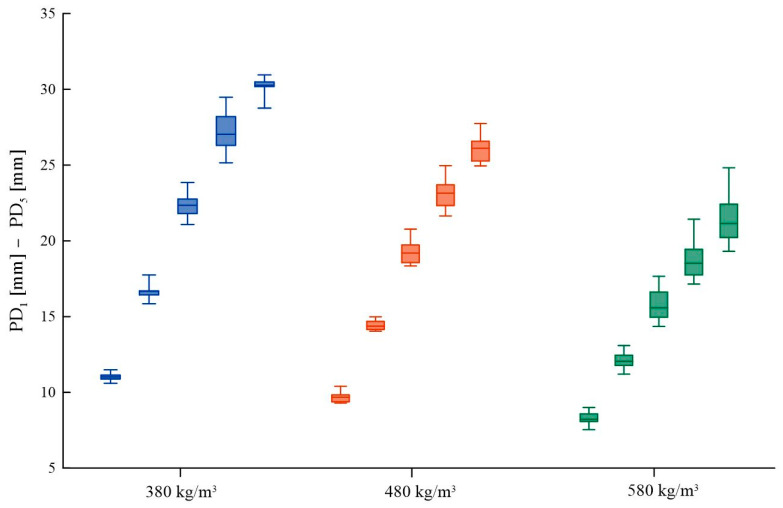
Sclerometric test results for 1 ÷ 5 impacts for different densities of fir wood.

**Figure 13:**
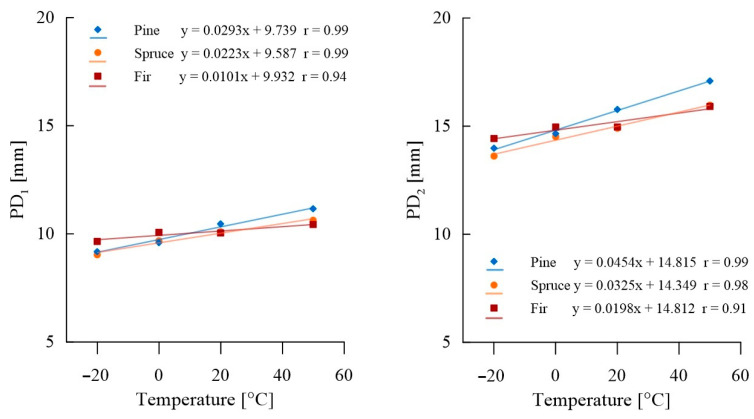
Sclerometric testing of samples at various temperatures.

**Table 1:** Compilation of changes in the depth of penetration of the sclerometer needle at different angles of testing relative to annual growth rings. Change in results relative to testing at a right angle to the growth rings expressed in percentages.

Angle Relative to Annual Growth Rings	PD_1_			PD_2_		
	Pine	Spruce	Fir	Pine	Spruce	Fir
60°	−2.86%	−2.16%	0.15%	−0.78%	−3.36%	0.36%
30°	1.59%	4.87%	5.97%	1.52%	1.36%	2.26%
0°	13.70%	9.21%	13.84%	7.20%	3.12%	8.17%

**Table 2:** Compilation of percentage changes in sclerometric test results for different orientations of the measuring device.

Orientation of the Device	PD_1_			PD_2_		
	380 kg/m^3^	480 kg/m^3^	580 kg/m^3^	380 kg/m^3^	480 kg/m^3^	580 kg/m^3^
vertically downward	−7.07%	−6.80%	−7.70%	−10.27%	−10.23%	−9.30%
vertically upward	5.61%	6.69%	7.89%	4.36%	5.90%	8.89%

**Table 3:** Compilation of percentage changes in sclerometric test results performed with multiple needle penetrations.

Material Density	PD_2_	PD_3_	PD_4_	PD_5_
380 kg/m^3^	51.6%	103.0%	145.5%	175.1%
480 kg/m^3^	48.7%	97.9%	138.5%	169.1%
580 kg/m^3^	46.8%	89.5%	125.4%	157.4%

**Table 4:** Compilation of percentage changes in the depth of penetration of the sclerometer needle for samples at various temperatures. Percentage change in results for every 10 °C change in temperature.

Test Temperature	PD_1_			PD_2_		
	Pine	Spruce	Fir	Pine	Spruce	Fir
+50 °C	6.64%	5.16%	3.82%	8.28%	7.08%	6.39%
0 °C	−8.23%	−4.25%	−0.2%	−7.11%	−2.69%	−0.1%
−20 °C	−12.2%	−10.57%	– 3.92%	−11.36%	−8.74%	−3.55%
average for a change of ±10 °C	3.01%	2.33%	1.02%	3.06%	2.26%	1.34%

**Table 5:** Value of the correction coefficien *r* relating to percentage changes in sclerometric testing for different testing directions relative to annual growth rings.

Angle Relative to Annual Growth Rings	PD_1_			PD_2_		
	Min	Mean	Max	Min	Mean	Max
60°	−0.02	−0.01	0.001	−0.03	−0.01	0.004
30°	0.01	0.04	0.05	0.01	0.01	0.02
0°	0.09	0.12	0.14	0.03	0.06	0.08

**Table 6:** Value of the coefficient *p* relating to percentage changes in sclerometric test results for different orientations of the measuring device.

Test Temperature *X_t_*	PD_1_	PD_2_
vertically downward	−0.07	−0.1
vertically upward	0.07	0.07

**Table 7:** Value of the coefficient *m* relating to percentage changes in sclerometric test results for a greater number of sclerometer impacts.

	PD_x_			
	PD_2_	PD_3_	PD_4_	PD_5_
m	0.5	0.95	1.35	1.65

**Table 8:** Value of the coefficient *u* relating to percentage changes in sclerometric test results for temperatures other than the reference temperature.

Test Temperature *X_t_*	Min	Mean	Max
higher than 20 °C	−0.003	−0.002	−0.001
lower than 20 °C	0.003	0.002	0.001

## Data Availability

Data are contained within the article and Appendix A.

## Appendix A

**Table A1:** Results of sclerometric tests conducted at different angles relative to annual growth rings.

		Pine				Spruce				Fir			
		x_min_	x_mean_	x_max_	s	x_min_	x_mean_	x_max_	s	x_min_	x_mean_	x_max_	s
**PD_1_** [mm]	90°	9.53	**11.01**	11.65	0.75	10.12	**11.52**	12.27	0.70	10.00	**10.51**	11.58	0.58
	60°	9.57	**10.70**	11.53	0.76	10.70	**11.27**	11.95	0.47	9.74	**10.52**	11.53	0.60
	30°	9.80	**11.19**	12.35	0.91	10.66	**12.08**	13.46	1.05	9.12	**11.14**	13.05	1.51
	0°	11.00	**12.52**	14.20	1.13	10.63	**12.58**	14.40	1.29	10.45	**11.96**	13.61	1.00
**PD_2_** [mm]	90°	14.93	**15.88**	16.65	0.67	15.83	**16.71**	17.80	0.77	14.72	**15.34**	16.23	0.48
	60°	14.77	**15.76**	16.68	0.80	15.09	**16.15**	17.12	0.77	14.50	**15.40**	16.20	0.60
	30°	14.80	**16.12**	17.19	0.79	15.56	**16.94**	18.36	0.98	14.55	**15.69**	17.25	1.08
	0°	15.65	**17.02**	18.96	1.05	14.44	**17.23**	19.40	1.73	15.15	**16.60**	17.98	0.90

**Table A2:** Results of sclerometric tests conducted for different orientations of the measuring device.

	Horizontal Testing		Vertical Downward Testing		Vertical Upward Testing	
	PD_1_ [mm]	PD_2_ [mm]	PD_1_ [mm]	PD_2_ [mm]	PD_1_ [mm]	PD_2_ [mm]
380 [kg/m^3^]						
x_min_	10.60	15.85	11.47	17.76	10.00	15.37
**x_mean_**	**11.00**	**16.68**	**11.78**	**18.39**	**10.38**	**15.95**
x_max_	11.49	17.75	12.13	19.17	10.70	16.62
s	0.25	0.44	0.19	0.38	0.24	0.35
CV	2.28	2.64	1.64	2.08	2.34	2.17
480 [kg/m^3^]						
x_min_	9.32	14.04	9.81	15.12	8.47	12.95
**x_mean_**	**9.68**	**14.40**	**10.34**	**15.87**	**9.03**	**13.55**
x_max_	10.40	14.99	10.88	16.51	9.72	14.26
s	0.29	0.32	0.28	0.47	0.37	0.41
CV	2.95	2.23	2.68	2.97	4.09	3.04
580 [kg/m^3^]						
x_min_	7.54	11.20	7.90	12.11	6.68	10.00
**x_mean_**	**8.25**	**12.11**	**8.88**	**13.23**	**7.60**	**11.03**
x_max_	9.00	13.09	9.78	14.22	8.55	12.20
s	0.42	0.54	0.49	0.58	0.55	0.54
CV	5.05	4.46	5.51	4.37	7.28	4.93

**Table A3:** Results of sclerometric tests performed with multiple needle penetrations.

	PD_1_ [mm]	PD_2_ [mm]	PD_3_ [mm]	PD_4_ [mm]	PD_5_ [mm]
380 [kg/m^3^]					
x_min_	10.60	15.85	21.08	25.15	28.76
**x_mean_**	**11.00**	**16.68**	**22.33**	**27.00**	**30.26**
x_max_	11.49	17.75	23.85	29.48	30.95
s	0.25	0.44	0.82	1.12	0.48
CV	2.28	2.64	3.69	4.15	1.58
480 [kg/m^3^]					
x_min_	9.32	14.04	18.35	21.65	24.94
**x_mean_**	**9.68**	**14.40**	**19.15**	**23.08**	**26.05**
x_max_	10.40	14.99	20.78	24.96	27.75
s	0.29	0.32	0.71	1.01	0.89
CV	2.95	2.23	3.71	4.39	3.40
580 [kg/m^3^]					
x_min_	7.54	11.20	14.35	17.15	19.31
**x_mean_**	**8.25**	**12.11**	**15.63**	**18.59**	**21.23**
x_max_	9.00	13.09	17.66	21.43	24.82
s	0.42	0.54	0.96	1.21	1.48
CV	5.05	4.46	6.17	6.48	6.95

**Table A4:** Results of sclerometric tests conducted at samples at various temperatures.

		Pine				Spruce				Fir			
		x_min_	x_mean_	x_max_	s	x_min_	x_mean_	x_max_	s	x_min_	x_mean_	x_max_	s
**PD_1_** [mm]	−20	8.55	**9.19**	10.35	0.50	8.30	**9.04**	10.67	0.84	8.24	**9.66**	11.42	1.05
	0	8.76	**9.60**	10.24	0.43	8.70	**9.68**	12.28	1.00	8.48	**10.08**	11.32	0.87
	+20	9.43	**10.47**	11.56	0.79	8.84	**10.11**	11.42	0.85	9.13	**10.05**	11.10	0.64
	+50	10.75	**11.16**	11.97	0.36	9.98	**10.63**	11.80	0.57	9.05	**10.44**	11.18	0.69
**PD_2_** [mm]	−20	13.35	**13.99**	15.00	**0.45**	12.35	**13.61**	15.74	1.21	12.61	**14.42**	16.71	1.27
	0	13.65	**14.66**	15.50	**0.56**	13.33	**14.52**	17.83	1.29	13.28	**14.96**	16.35	0.91
	+20	14.07	**15.78**	16.96	**0.94**	13.63	**14.92**	16.68	1.18	13.96	**14.95**	16.05	0.75
	+50	16.35	**17.09**	18.00	0.47	14.86	**15.97**	17.26	0.71	14.52	**15.91**	17.78	1.00
